# Effect of Twinning on Angle-Resolved Photoemission Spectroscopy Analysis of Ni_49.7_Mn_29.1_Ga_21.2_(100) Heusler Alloy

**DOI:** 10.3390/ma15030717

**Published:** 2022-01-18

**Authors:** Vladimír Cháb, Václav Drchal, František Máca, Josef Kudrnovský, Stanislav Cichoň, Ján Lančok, Oleg Heczko

**Affiliations:** Institute of Physics, The Czech Academy of Sciences, Na Slovance 1999/2, 18221 Prague, Czech Republic; chab@fzu.cz (V.C.); drchal@fzu.cz (V.D.); maca@fzu.cz (F.M.); kudrnov@fzu.cz (J.K.); lancok@fzu.cz (J.L.); heczko@fzu.cz (O.H.)

**Keywords:** Heusler alloys, shape memory, band structure, photoemission spectroscopy, ferromagnetism, martensite, twinning

## Abstract

To explain the observed features of k-space photoelectron images taken on off-stoichiometric Heusler Ni_49_._7_Mn_29_._1_Ga_21_._2_ single-crystals in the cubic austenitic and pseudotetragonal martensitic phases, the images were simulated theoretically. Despite the moderate structural difference of both phases, there is large difference in photoemission spectra. Analysis of the final states’ structure, matrix elements, and interface barrier scattering was performed to interpret discrepancies between the external photoemission of the austenite and martensite. The missing signal at the surface-normal emission of the martensitic phase is, ultimately, explained by repeated scatterings of escaping electrons on the interfaces between nanotwins.

## 1. Introduction

A combination of ferroelastic and ferromagnetic ordering makes off-stoichiometric Ni-Mn-Ga Heusler alloys one of the most studied, functional materials [1,2]. The multiferroic properties arise after a particular, so-called martensitic, transition. The transition is a difussionless, solid-state transformation from cubic austenite to the lower symmetry martensite phase in a ferromagnetic state, resulting in the formation of ferroelastic ordering and respective ferroelastic domains by twinning. As the martensitic transformation in the off-stoichiometric compounds occurs above room temperature, it is relevant in real life [3]. To fully exploit the giant, field-induced deformation, called the magnetic shape memory effect, and similar phenomena, we have to handle not only ferroelastic ordering or twinning and magnetic domains’ structure, but also the occupation of minority or majority spins in particular parts of a crystal and tailor the electronic structure [4,5,6]. Most of the studies of these functional materials were done on the crystal twinning and related structure of magnetic domains and determination of their ferromagnetic and ferroelastic properties [7,8]. Electronic structure was treated reasonably with theory showing a distribution of majority and minority spins in the valence bands in different phases. As a matter of fact, a real crystal in the martensitic phase was simplified, considering the crystal as a homogenous, bulk structure excluding all internal interfaces among twinning variants—ferroelastic domains [9,10].

There were a few attempts to determine electronic band structure experimentally either in the austenitic or martensitic phases. For both phases, the measurement of E vs. k dispersion relations was performed, fully covering the valence bands, followed by analysis concerning chemical and structural disorders. Generally, the band features were strongly smeared out, possessing DOS-like character in the relatively wide binding energy (BE) interval [11,12]. The theoretical analysis of the disorder was performed with expected sources, such as chemical disorder, due to alloying effect, i.e., off-stoichiometry and the structural defects resulting from the twinning [13]. However, the effect was not described successfully on the whole. In fact, the multiferroic structure of a sample formed with domains of particular magnetization and twin variants with different orientation must have influenced the electron transport of electrons with a magnetic gradient among them. This type of magnetic and structural scattering may be reflected in the photoemission process, as well as the spin-orbit splitting of the bands, due to their occupation with minority or majority spins.

In this paper, we demonstrate the difference in the photoemission spectra of electrons with minor and major spin polarisation in the valence band of the Ni_49.7_Mn_29.1_Ga_21.2_ alloy in the cubic austenitic and 10M, modulated, twinned martensitic phases, excited with an un-polarized, conventional light source. The experimental data are compared with theoretical calculations. Previous theoretical analysis was performed on the moderately disordered systems [11,12] with the classical photoemission three-step model. In order to explain the missing signal close to the $\bar{\Gamma }$ point, we analyze the final state effects up to 22 eV above the E_F_ and the scattering at interfaces among micro- and nanotwins and magnetic domain barriers. The observed, direction-dependent transmission is modelled using a multiple scattering of outgoing electrons on barriers arising from nanotwinning (regularly arranged stacking faults) in differently oriented, ferroelastic domains.

## 2. Materials and Methods

We used single-crystal Ni_49_._7_Mn_29_._1_Ga_21_._2_ with a transformation temperature from cubic austenite to martensite of above RT. The martensite phase is a modulated, 10M structure with a monoclinic unit cell. Considering the resolution of photoemission spectrometer, the martensite structure can be well approximated as a (pseudo)tetragonal structure with c = 0.565 nm and a ≅ b = 0.596 nm. The short c-axis is an easy axis of magnetization. Ferromagnetic Curie temperature is 373 K. The crystal exhibits magnetism-induced twin variant reorientation, resulting in giant strain in magnetic field below 1 T.

An analyzed sample was cut along the (100) plane of austenite with precision of a few degrees. The sample was electropolished at the martensite phase and prepared for ultra-high vacuum (UHV) by cycles of Ar^+^ sputtering and subsequent annealing at 400 °C prior to each measurement. In addition to slight miscut, the (100) and (001) planes deviated further from the surface plane by a few degrees due to the geometry of transformation and twinning [14,15]. The detailed description of the experimental conditions is given in reference [16].

NanoESCA photoemission spectrometer (Scienta Omicron GmbH, Taunusstein, Germany) based on a photoelectron emission microscopy (PEEM) column and double hemispherical imaging energy filter was used. The basic pressure in the analytical chamber was lower than 10^−8^ Pa. The surface quality was controlled with the low energy electron diffraction (LEED) and the electron spectroscopy for chemical analysis (ESCA) mode of the spectrometer. The PEEM microscope, operating in diffraction mode, was used to acquire the equi-energetic cuts (EEC) through the first Brillouin zone (BZ) using a He-I discharge lamp.

## 3. Results

### 3.1. Experiment

**Figure 1:**
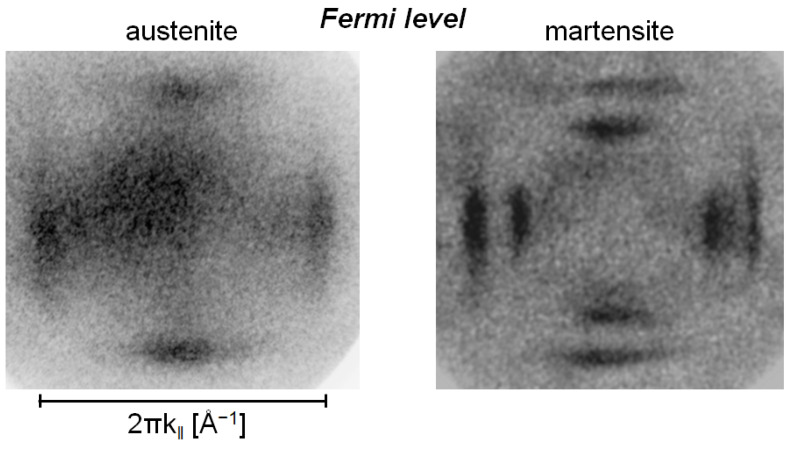
Equi-energy cuts (EEC) at Fermi level of austenite and martensite phase of Ni_49_._7_Mn_29_._1_Ga_21_._2_.

**Figure 2:**
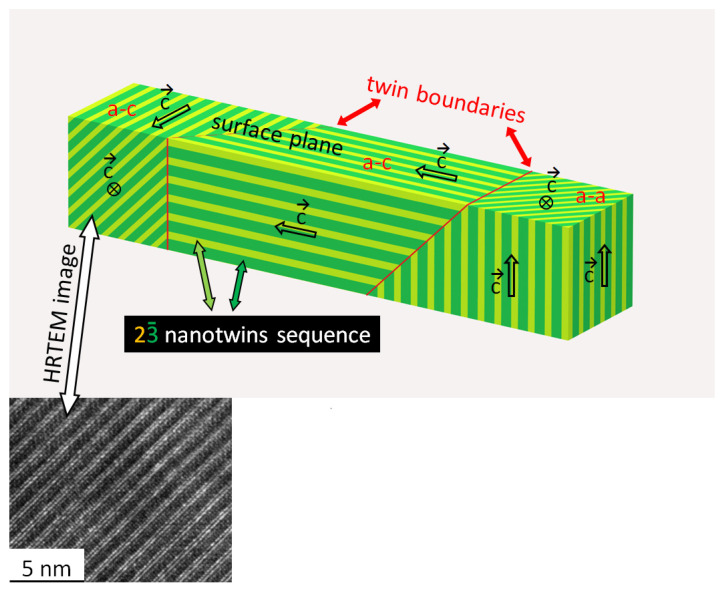
Schematic depiction of (32) nanotwinning in martensite; two a-c variants and one a-a variant are marked. Other variants can be obtained by rotation. High resolution transmission electron microscopy (HRTEM) cross-section image shows arrangement of the nanotwins.

The EECs through the first BZ of the Ni_49_._7_Mn_29_._1_Ga_21_._2_ sample in the austenitic and martensitic phases were published in our previous papers [11,12,16]. Typical examples of the energy cuts at E_F_ of austenite and martensite are shown in Figure 1. The austenite showed a regular, symmetrical pattern reflecting cubic symmetry. In the martensitic phase, the observed splitting of the bands close to the BZ border was interpreted as a result of the superposition of different electronic structures arising from ferroelastic ordering, i.e., twinning [12]. Spontaneous martensitic transformation usually results in many differently oriented twin variants [14,15]. In the case of Ni-Mn-Ga, considering our pseudotetragonal approximation and (100) surface plane orientation, there were three different ferroelastic domains or twin variants: one a-a variant having crystal a-axes in and a c-axis perpendicular to the surface plane and two a-c variants with a- and c-axes in and an a-axis perpendicular to the surface plane. The presence of two a-c variants resulted in the observed splitting in the photoemission image [12]. For variant visualization we referred to the schematic 3D model of the twinned microstructure shown in Figure 2.

In addition to the band splitting due to the twinned microstructure, the comparison of the ECC of austenite and martensite in Figure 1 shows a surprising fact: that the martensitic phase exhibits weak or no electron emission in its central part, i.e., there is no photoelectron signal around the $\bar{\Gamma }$ point, in contrast to the austenitic cubic phase. Moreover, theoretical 3D calculations of martensite also predict the photoelectron emission close to the $\bar{\Gamma }$ point [17,18].

The electron DOS of minority and majority spins located in the valence bands demonstrated that electrons with majority spin form mostly the outer square features, while interior features around the center of the surface Brillouin zone (SBZ) are formed by minority spin electrons. Thus, the experimental EEC reflected both features since the excitation light was not polarized and detected electrons were not spin-resolved in our experiment. Figure 1 shows clearly the expected contribution of electrons with both spins in the austenitic phase, while the EEC of the martensitic one consisted predominantly of the contribution from the majority spin electrons and no or weak emission from minority spin electrons.

Moreover, comparison of the experimental EECs from different stages of the austenite–martensite phase transition shows that the photoemission from the minority spin polarized electrons was observed in the presence of perpendicularly oriented magnetization in martensite and with magnetization in plane in austenite [12]. This indicates that the magnetization orientation is not a decisive factor in reducing intensity of the photoemission in the $\bar{\Gamma }$ point. Previous measurement identified the missing emission from minority spin polarization at cuts below 2 eV BE. In the cuts taken at 2.0 eV and 2.1 eV BE, the reverse effect was observed: they reproduced the projection of minority spin DOS on the a-a face only and showed no majority spin contribution [12]. To explain the observed disparities, we consider several theoretical options dealing with changes of electronic structure and crystal microstructure.

### 3.2. Theory

The angle-resolved photoemission spectroscopy (ARPES) intensity was calculated in the same way as in [11,12]. We approximated the semi-infinite solid by a bulk phase, employed the approximation of a constant matrix element, and neglected the final-state effects. We employed the tight-binding, muffin-tin orbital method and the alloy randomness was included via the coherent-potential approximation (CPA) [13]. Thus, the information on the electronic structure and Bloch spectral density was contained in the configurationally averaged Green function. Instead of the BZ, we used a prismatic zone with the same volume as the BZ, and its repetition covered the entire reciprocal space. The k-vector was decomposed into its parallel k_‖_ (with the surface) and perpendicular k_z_ parts. We divided the basal plane of the prismatic zone into small rectangles with sides equal to 2π/(80a) or 2π/(80c) that defined the k-space sampling.

**Figure 3:**
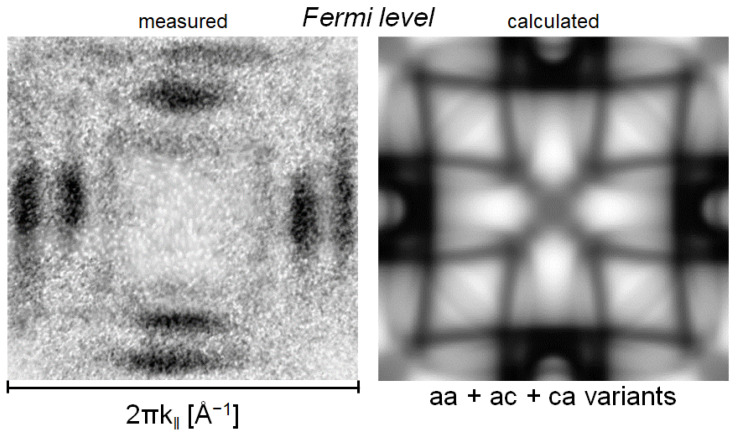
Photoemission intensity from martensitic phase at Fermi level measured (**left**) and calculated (**right**). The displayed intensities are relative quantities. Therefore, they do not allow a direct quantitative comparison between experiment and theory. The theoretical intensity is taken from Ref. [12].

EEC was calculated for BE between E_F_ and E_F_—3 eV [12]. Within the EEC’s theoretical simulation of the martensitic phase, the twinning microstructure and the arrangement of the experiments were taken into account. EECs excited with un-polarized light (21.28 eV) for the sample with combined a-a and a-c twin variant surfaces are shown in the Figure 3. Separate simulations for the a-a and a-c variants showed significant contributions to the photoemission. Photoelectrons from both spin orientations were present in the EEC, contrary to the experimental spectra in which weak external emission close to the $\bar{\Gamma }$ point was detected.

To summarize the results of simulation, we found photoemission at the $\bar{\Gamma }$ point due to the contribution of majority spin DOS attributed to the a-c face. To explain this discrepancy, we analyzed the final-state effects and the effect of the martensitic microstructure.

**Figure 4:**
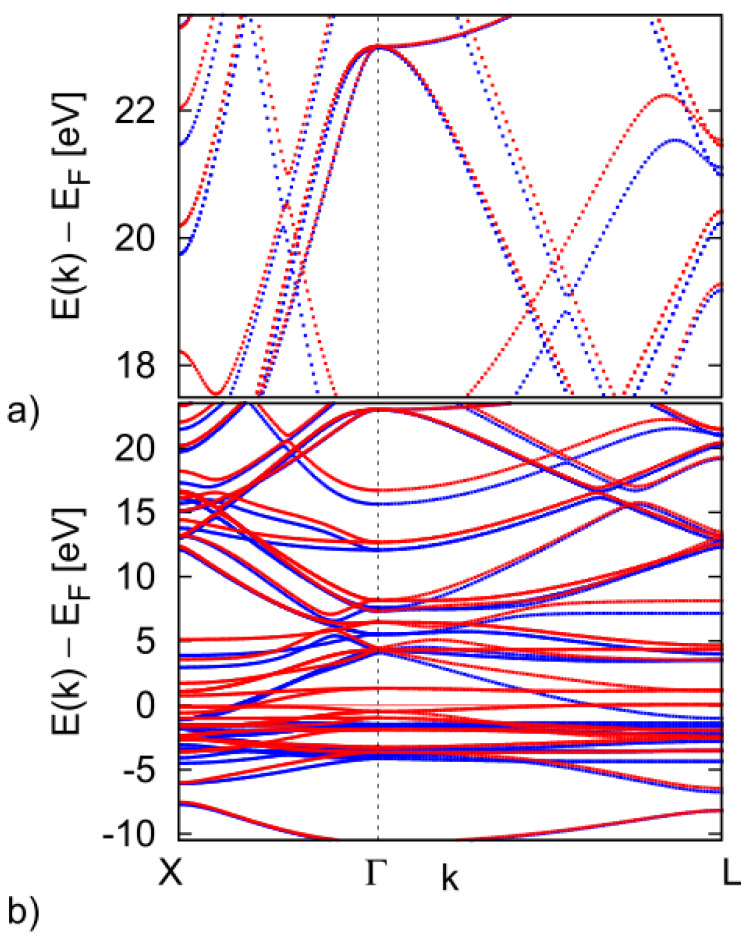
Spin-resolved band structure of austenite phase of Ni_2_MnGa. (**a**) Detail of energy range around the value of excitation radiation energy, 21.28 eV, employed for experimental measurements. (**b**) Band structure in the larger energy range. Red lines denote minority spin bands while blue lines denote majority spin bands.

First, we checked the final-state effects. The effect was considered in the frame of the three-step model [19]. Our slightly disordered system was not eligible for inverse LEED approximation. Therefore, we calculated the electronic band structure of crystalline Ni_2_MnGa up to energies 22 eV above E_F_ to check the final-state bands [20]. The bands were calculated first for the austenitic phase to test the presence of gaps at the Γ point approximately at ~18 eV above the E_F_. A full Heusler structure with four FCC lattices shifted by a ¼ of the space diagonal was considered (space group no. 216, F$\bar{4}$3 m). The experimental lattice parameter a = 0.5825 nm was used in VASP calculations [21]. The spin polarized calculations, as well as calculations including spin orbital interaction, were performed using the projector augmented wave scheme. We used the GGA exchange correlation potential, 752 k-points, and 48 symmetry operations, E_cut_ = 300 eV. The difference between input and output charge densities in the self-consistent procedure was <0.1 me bohr^−3^. The results for the relaxed, magnetic, stoichiometric Ni_2_MnGa crystal in the austenitic phase are displayed in Figure 4 for X-Γ-L symmetry directions. The escaping electrons could move freely in any direction as there was no gap. This is seen from Figure 4 because the state carrying electrons in the direction (0, 0, k_z_) exists on the segment Γ-X, where X = 2π (0, 0, 1)/a. We note that the gap visible in Figure 4 at around 20 eV in the 3D band structure in the vicinity of the Γ point disappears in the projection of the 3D band structure into two dimensions (the surface 2D BZ). The situation is similar in the case of martensite. Therefore, in both structural modifications, the final states cannot considerably influence the spectra.

Second, the effect of the matrix element can hardly make distinction between the austenite and martensite because there is no reason for substantial difference of matrix elements between these two crystallographic modifications. Their change for martensitic and austenitic structures is trivial and it cannot open or close a channel for the external photoemission.

As there is no theoretical reason for the observed differences, considering electronic structure, we have to take into account the possible consequences of microstructural or, rather, nanostructural imperfections. The escape depth for photoelectrons generated by the 21.28 eV radiation in metals such as Fe or Cu, i.e., close to Ni, Mn, and Ga, should be around 8.0 to 15.0 Å [22,23,24]. More precise values cannot be provided as the most recent works reported on a large scatter in values for low energy electrons. Under these conditions, the wavelength of the escaping electrons was approximately 3.0 Å, being comparable to the interatomic distance. Consequently, this rules out the effect of magnetic domains, the typical size of which is around 1 μm, in agreement with the experiment discussed previously. The comparably small escape depth also rules out the macroscopic a/c twinning which occurs spontaneously after martensitic transformation on a much larger scale [25]. Based on the same argument, we can also exclude a/b and modulation twins that are present in real 10M structure [26,27], albeit not in our pseudotetragonal approximation.

However, we should consider that the 10M martensite structure is modulated. The modulation can be considered as a regular arrangement of stacking faults or nanotwins of two and three atomic layers [28,29,30,31,32]. Given the size of the Ni_49.7_Mn_29.1_Ga_21.2_ Heusler unit cell, ~6.0 Å, comparable to the separation between individual nanotwin interfaces in the modulated structure and the values of electron escape depth, >8.0 Å, these interfaces [33] are the most probable candidates for the scattering.

Figure 2 shows the orientation of these layered nanotwins in relation to macroscopic a/c twinning resulting in a-a and a-c twin variants together with an HRTEM micrograph. The micrograph clearly indicates such regularly arranged interfaces between nanotwins within a single a-c twin domain. From the symmetry, it follows that the a-c twin variants can have four different orientations of nanotwinning, although Figure 2 shows just two exemplary orientations. The a-c twin variants extend over a large volume and their nanotwinning structure can be effective scatterers. Thus, an interface between two nanotwins can be considered as a main source of scattering for passing electrons. Moreover, one can expect that the presence of these interfaces causes omnidirectional scattering leading to general smearing out and blurring of the photoelectron intensity.

**Figure 5:**
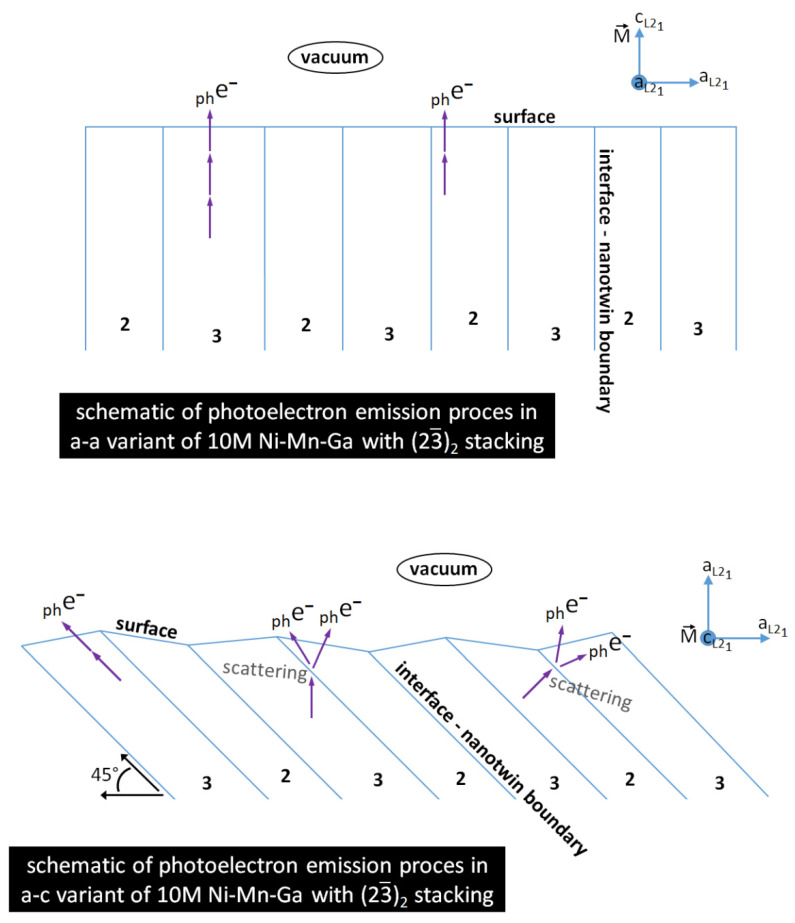
Scheme of photoelectrons’ trajectory during photoemission in martensite; comparison of a-a and a-c variants.

However, it is difficult to construct a faithful and precise model of electron transfer to the surface [34]. Instead, we can assume that the transmission through a rough interface leads to attenuation of the electron current in its original direction by a factor of *q* (*q <* 1). The rest of the electron wave is scattered in all other directions. After crossing *N* interfaces, the original intensity is reduced by a factor *q^N^*. The orientation of the electron velocity with respect to interfaces between nano-crystallites now becomes important: electrons moving parallel to interfaces are not scattered while those crossing several interfaces can be severally damped. This scattering and emission process is drawn schematically in Figure 5.

In the model, only a-c twin variants are considered, which are only possible in the pseudotetragonal structure. In fact, as the real structure is monoclinic, there are other twinning modes which make the twinned microstructure quite complex [15,26,27,30,35]. The characteristic size of all these twinning structures is, however, much larger than the escape depth and, thus, this twinning cannot bring additional features to the spectra. Despite the twinning complexity of real 10M martensite structure, there are only four inclined directions of modulation and, thus, four symmetrical orientations of the interfaces on which the electrons scatter. Two of them are drawn in Figure 2.

## 4. Discussion

**Figure 6:**
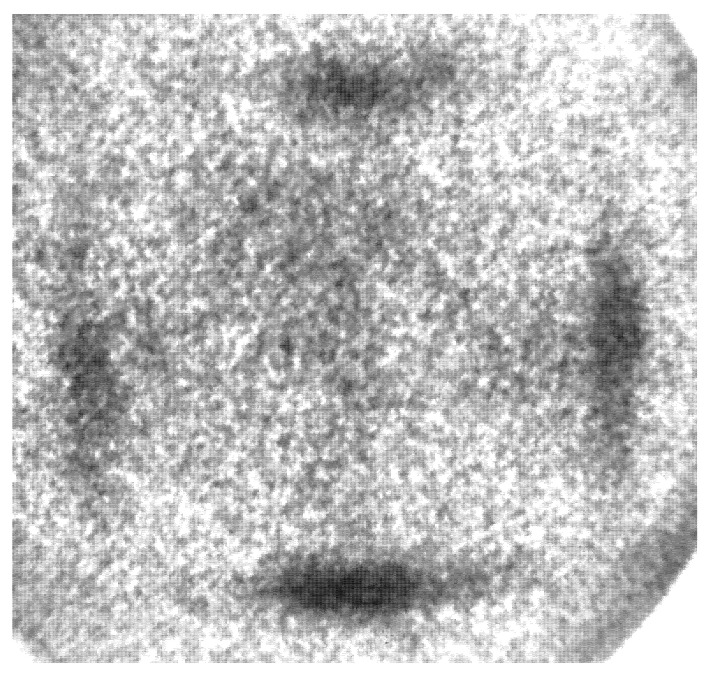
Equi-energy cut at Fermi level of single a-a twin domain. The non-vanishing intensity around $\bar{\Gamma }$ point is apparent. The noisy nature of the image originates from an extremely small area available for analysis.

Our theoretical analysis and calculation showed that the difference between the observed intensities from austenite and from modulated 10M martensite is a consequence of electron scattering on particularly oriented interfaces between nanotwins or a regular arrangement of stacking faults present in the martensitic phase. These nanotwins are also often considered as modulation. The HRTEM image of these interfaces shown in Figure 2 clearly demonstrates the microscopic (2$\bar{3}$)_2_ arrangement. Such interfaces are not present in the cubic austenitic phase and, so, photoelectrons can freely leave, and the intensity is not influenced by an additional scattering. Although, in martensite, these nanotwin interfaces are always present, for the a-a variant, the interfaces are perpendicular to the surface and, thus, do not scatter. In particular, the intensity at the center and at the boundary of the SBZ should be visible for these cases. We were able to find a single martensitic twin area of ~10 μm in diameter where the emission round $\bar{\Gamma }$ point was observed (see Figure 6). The symmetry of the pattern and no splitting on the edges demonstrated that the measurement was performed on symmetrical a-a twin domain. In such a case, the electron has an interface free path to the surface (Figure 5) and the emission around the $\bar{\Gamma }$ point arises.

In a-c variants, the interfaces form the angle 45° with the surface plane (001). The electrons moving in the direction perpendicular to the surface (and contributing to intensity in the $\bar{\Gamma }$ point) are damped, while those moving in a skew direction (close to 45°) are damped much less. This can explain why the intensity around the center of the SBZ vanishes and the intensity at the SBZ boundary is preserved much better. This effect of direction-dependent transmission is sketched in Figure 5 where the most important, a-c, domain orientation is stressed.

## 5. Conclusions

We compared ARPES intensities measured on off-stoichiometric, thus partially disordered, single-crystals of Ni_49_._7_Mn_29_._1_Ga_21_._2_ in the austenitic and in the martensitic phases. A favorable agreement was found, with intensities calculated from first principles for austenite, while there were deviations in the martensitic phase. The main deviation was that the intensity in the center of the SBZ was missing.

We show that the final state’s effect cannot reduce photoemitted electrons to zero for the martensitic phase. Analysis of the matrix elements led to a similar conclusion. Among various mechanisms considered, we found that the main cause is the scattering of photoelectrons on interfaces between nanotwins present in the modulated 10M martensite in particularly oriented twin (ferroelastic) domains. The presence of multiple, inclined interfaces in the modulated martensitic phase reduces the external photoemission along the normal of the surface.

## Data Availability

Not applicable.
